# Doppler ultrasound, a noninvasive tool for the study of mesenteric arterial flow in systemic sclerosis: a cross-sectional study of a patient cohort with review and meta-analysis of the literature

**DOI:** 10.1007/s11739-024-03783-5

**Published:** 2024-10-16

**Authors:** Giulia Bandini, Matteo Monami, Gabriele Ciuti, Paolo Mercatelli, Anna Lo Cricchio, Maria Cristina De Santis, Francesco Bonomi, Silvia Bellando Randone, Corrado Campochiaro, Khadija El Aoufy, Barbara Ruaro, Dilia Giuggioli, Michael Hughes, Zsuzsanna H. McMahan, Devis Benfaremo, Gianluca Moroncini, Giovanni Maconi, Esterita Accogli, Lorenzo Dagna, Marco Matucci Cerinic, Alberto Moggi Pignone

**Affiliations:** 1 Department of Experimental and Clinical Medicine, Division of Internal Medicine, University of Florence, Careggi Hospital, Largo Brambilla 3, Florence, Italy; 2 Azienda Ospedaliero Universitaria Careggi and University of Florence, Florence, Italy; 3 Department of Experimental and Clinical Medicine, Division of Rheumatology, University of Florence, Careggi Hospital, Florence, Italy; 4 Unit of Immunology, Rheumatology, Allergy and Rare Diseases (UnIRAR), IRCCS San Raffaele Hospital, Milan, Italy; 5 Department of Experimental and Clinical Medicine, University of Florence, Florence, Italy; 6 Department of Medical Surgical and Health Sciences, Pulmonology Unit, University Hospital of Cattinara, University of Trieste, Trieste, Italy; 7 Rheumatology Unit, University Hospital of Modena and Reggio Emilia School of Medicine Department of Medical and Surgical Sciences for Children and Adults, Reggio Emilia, Italy; 8 Northern Care Alliance NHS Foundation Trust, Salford Care Organisation, Salford, UK; 9 Division of Musculoskeletal and Dermatological Sciences, The University of Manchester, Manchester Academic Health Science Centre, Manchester, UK; 10 Division of Rheumatology, Department of Medicine, UTHealth Houston, Houston, USA; 11 Department of Clinical and Molecular Sciences, Marche Polytechnic University, Ancona, Italy; 12 Department of Internal Medicine, Marche University Hospital, Ancona, Italy; 13 Gastroenterology Unit, Department of Biomedical and Clinical Sciences, ASST Fatebenefratelli-Sacco, University of Milan, Milan, Italy; 14 Department of Internal Medicine, Centre of Research and Learning in Ultrasound, Maggiore Hospital, Bologna, Italy

**Keywords:** Systemic sclerosis, Scleroderma, Bowel vasculopathy, Superior mesenteric artery, Inferior mesenteric artery, Pathogenesis, Outcome measures

## Abstract

Gastrointestinal involvement (GI) is a frequent and troublesome complication of systemic sclerosis (SSc), whose etiology is poorly understood, though it is hypothesized that autoimmunity and progressive vasculopathy may play a role. Vasculopathy is considered one of the main pathogenetic pathways responsible for many of the clinical manifestations of SSc, and, therefore, studying the principal splanchnic vessels (i.e., superior mesenteric artery—SMA and inferior mesenteric artery—IMA) with Doppler Ultrasound (DUS) may provide further insights into measuring the progression of vasculopathy, evaluating its possible association with SSc GI symptoms, and determining whether it plays a role in the development or severity of SSc GI disease. A cohort of SSc patients consecutively recruited underwent DUS examination, and associations with GI (UCLA-GIT 2.0 questionnaire) and extraintestinal SSc characteristics were evaluated. Semiquantitative DUS parameters (resistive index—RI and pulsatility index—PI), were applied for splanchnic vessel assessment in SSc patients and healthy subjects (HS). Moreover, a review and meta-analysis of the literature to understand which the values of the main semiquantitative DUS parameters (RI and PI) are both in SSc patients and HS has been conducted. Seventy-eight patients completed DUS examinations and clinical assessments. 30 (39%) were classified as diffuse cutaneous SSc (dcSSC), 35 (45%) as limited cutaneous SSc (lcSSc) and 13 (17%) as sine scleroderma. A significant difference was found both for SMA RI (*p* for trend = 0.032) and SMA PI (*p* for trend = 0.004) between patients with sine scleroderma, lcSSc and dcSSc, with lower values observed in the sine scleroderma and lcSSc groups. IMA RI and PI were significantly correlated with GI symptoms such as fecal incontinence (*ῥ* − 0.33, *p* = 0.008 and *ῥ* − 0.30, *p* = 0.021, respectively). By multivariate analysis, significant associations were confirmed between SMA RI and SMA PI and mRSS (*β* 0.248, *p* = 0.030 and *β* 2.995, *p* = 0.004, respectively) and with bosentan (*β* 0.400, *p* = 0.003 and *β* 3.508, *p* = 0.001, respectively), but not with anticentromere antibody (ACA). No significant differences were found between the weighted median values of SMA RI and SMA PI of SSc patients compared to those of HS that were derived from the meta-analysis of the literature (*p* = 0.72 and *p* = 0.64, respectively). This cross-sectional study confirms that the splanchnic vasculature of SSc patients can noninvasively been studied with DUS. Vascular splanchnic involvement correlates with the presence and/or severity of specific clinical features in SSc, including GI. Larger and prospective studies are needed to confirm these preliminary observations and to examine the role of DUS in SSc-risk stratification and GI progression and to obtain definitive data regarding both HS and SSc patients splanchnic DUS parameters.

**Supplementary Information:**

The online version contains supplementary material available at 10.1007/s11739-024-03783-5.

## Introduction

Systemic sclerosis (SSc) is a rare, chronic autoimmune disease characterized by a complex pathophysiology involving humoral and cellular immunity, endothelial cells and fibroblasts leading to widespread vascular injury and excessive deposition of collagen fibers in the skin and major internal organs [1, 2].

The gastrointestinal (GI) tract is one of the most frequently involved organs, affecting the majority (75–90%) of SSc patients [3]. The GI manifestations are heterogeneous as every region, from the esophagus to the anorectum, may be involved [4]. In the early phases, GI involvement is often largely asymptomatic [5] because it is frequently subclinical until severe tissue damage occurs [3, 6]. Once symptoms occur, SSc-related GI involvement heavily impacts morbidity and mortality, with patients experiencing significant emotional and social burdens and decreased survival in severe cases [7]. Symptoms may include heartburn, dysphagia, abdominal distension, weight loss, diarrhea, fecal incontinence, and constipation [5, 8]. Upper GI involvement can lead to esophageal dysmotility, gastroesophageal reflux disease, lower esophageal sphincter dysfunction, gastroparesis, and even GI bleeding culminating in esophagitis, esophageal or gastric ulcers, and gastric antral vascular ectasia [9]. Moreover, the esophageal dysfunction is tightly associated with interstitial lung disease [10, 11]. Lower GI-tract involvement (i.e., small bowel, large bowel, and/or anorectal) can also be quite severe, with complications including small intestinal bacterial overgrowth (SIBO), intestinal pseudo-obstruction, intestinal pneumatosis and fecal incontinence [12–14].

Early recognition of GI involvement is pivotal for the control of symptoms as well as learning how to prevent complications. Unfortunately, the diagnosis of GI involvement is challenging because it is often delayed by the paucity of signs and symptoms early in the disease. Moreover, several diagnostic investigations that are useful in defining GI involvement are either invasive, expensive or not very informative [15].

The progression of vascular disease is known to result in the damage of many tissues impacted by SSc, such as the digital and pulmonary vasculature. However, the ability to easily measure vascular disease activity remains a major challenge in patients with SSc and limits the early application of targeted therapies. Understanding the etiology and early phases of GI involvement, therefore, remains a high priority in SSc research. The aim would be to identify patients at high risk of developing progressive GI involvement, monitor disease activity, in the vessels or other relevant tissues, and intervene before the development of significant damage/dysfunction.

Abdominal ultrasound (US) is a unique tool that allows for the noninvasive assessment of the vasculature and major parts of the GI tract, including extra-intestinal features and splanchnic vessels [16]. Although US is often utilized in the investigation of the musculoskeletal system, and now also of the lung [17], its role in the study of SSc-related vascular and GI disease has not been fully considered.

Clinical observations and basic studies suggest that microvascular vasculopathy is of paramount importance throughout all the phases of SSc. However, increasing evidence also implicates macrovascular involvement as possibly playing a key role in the disease [18].

To our knowledge, only a few studies have evaluated splanchnic circulation in SSc. In 2002 [19], some differences in Doppler Ultrasound (DUS) parameters were noted between patients and controls, even in the absence of symptoms of small bowel involvement. In 2005, the effect of a prostacyclin analog in peripheral circulation of patients with SSc, including mesenteric arteries, was explored [20]. In 2022 a difference in some of the main DUS parameters between patients and controls, both for superior mesenteric artery (SMA) and inferior mesenteric artery (IMA) was detected [21]. All these studies included small numbers of patients, and consequently, it was not possible to evaluate any correlations between the DUS parameters and the patient’s demographic, clinical, and organ involvement characteristics. Furthermore, the comparison of DUS parameters between patients and controls was conducted in small numbers of subjects and was therefore also hard to interpret.

To our knowledge, the normal fasting flow of the SMA has been analyzed in studies where healthy subjects (HS) were present as a control group, except for some recent papers [22]. While many of these papers report normality data on the resistive index (RI), few data about normality values of the pulsatility index (PI) are available. Even fewer data are available to determine the normal fasting flow of the IMA. IMA is a small-caliber vessel (2–3 mm), not always easy to study with DUS given its small size and the less than favorable viewing angle given the position relative to the abdominal aorta.

Semiquantitative Doppler parameters (i.e., RI and PI) provide fundamental hemodynamic information of the explored vascular district, being correlated to vascular resistance and compliance. Resistive index, the difference between peak systolic velocity (PSV) and end diastolic velocity (EDV) divided by PSV, varies from 0 to 1. Higher values indicate greater resistance to blood flow in the bed vascular downstream of the measurement point. It typically increases as a vessel narrows (i.e., a stenotic vessel) indicating an increase in resistance to blood flow. PSV is the value of the maximum systolic velocity (cm/sec), EDV is the value of the EDV (cm/sec), while time-averaged maximum velocity (TAMV) is the average speed value of the entire cycle. Pulsatility index, which is the difference between PSV and EDV divided by TAMV, is strongly related to elasticity of arterial vessels and it has been used in many different clinical scenarios as a measure of compliance and resistance of arterial blood flow. Because PI also includes median velocity it is considered to be better related to the cardiac cycle. Moreover it provides information about the vessel’s ability to dampen the flow from systole to diastole and is inversely related to vascular elasticity. They both offer well-known advantages, foremost among them being that they represent a semiquantitative value rather than an absolute value. This avoids potential errors in estimating absolute velocities, the measurement of which is closely tied to the Doppler incidence angle.

Therefore, the aim of the present work was first to confirm, in a larger sample of SSc patients, the previous data, noninvasively studying the splanchnic circulation with DUS (i.e., both SMA and IMA) and to determine whether DUS parameters are associated with the presence and severity of SSc clinical features, including GI disease.

At the same time, to perform a review of the literature to better understand which normal semiquantitative DUS parameters should be considered when studying mesenteric arteries blood flow and compare them with those obtained in SSc patients.

## Methods

### Review of the literature and meta-analysis

#### Search strategy and selection criteria

The present analysis included all observational studies assessing mesenteric arterial blood flow using DUS, including HS and patients with SSc. We included studies in the English language with no date restriction. A Medline and Embase search was performed up to February 1st, 2024 using the following search string: mesenteric artery and ultrasound. Detailed information on the search strategy is reported in Table S1. Further studies were manually searched for references from retrieved papers.

#### Selection criteria

To be eligible, a study should enroll patients with SSc or HS with the assessment of the SMA and/or the IMA flow with DUS.

Two independent reviewers (ALC and MCDS) screened all titles and abstracts of the identified studies for inclusion. Discrepancies were resolved by a third, independent reviewer (GB).

#### Data extraction and collection

Variables of interest were age, gender, number of included subjects, SMA resistive index (RI), SMA pulsatility index (PI), IMA RI, and IMA PI.

Data extraction was performed independently by two of the authors (GC and PM), and conflicts were resolved by a third investigator (GB).

Titles and abstracts were screened independently by the authors, and potentially relevant articles were retrieved in full text. For all published studies, results reported in published papers and supplements were used as the primary source of information. The identification of relevant abstracts and the selection of studies were performed independently by all the authors.

#### Endpoints

The primary endpoint was the definition of median values (interquartile) for SMA RI and PI among HS and SSc patients. Secondary endpoint was the definition of median values (interquartile) for IMA RI and PI among HS and SSc patients. Other secondary endpoints were the correlation of SMA RI and PI with age and gender in HS.

## Cohort study

### Patients

Patients were classified as SSc based on ACR/EULAR 2013 classification criteria. At the time of the US examination, the following demographic, clinical and organ involvement information, available from the routine clinical assessment were collected from clinical charts: age and sex; disease subset (i.e., sine scleroderma, limited cutaneous systemic sclerosis—lcSSc, diffuse cutaneous systemic sclerosis—dcSSc), disease duration; modified Rodnan Skin Score (mRSS); nailfold capillaroscopy (NVC) patterns (not specific, early, active, late); presence or absence of digital ulcers (DUs); laboratory values (i.e., erythrocyte sedimentation rate—ESR, C-reactive protein—CRP, creatinine clearance), including autoantibodies profile (anti-Scl70 and ACA positive); spirometry parameters (i.e., forced vital capacity—FVC, forced expiratory volume in the first second—FEV1, diffusing capacity of the lungs for carbon monoxide—DLCO); echocardiographic data (i.e., ejection fraction—EF, pulmonary artery pressure—PAPs); renal artery resistive index (RRI); data regarding therapy.

Every patient completed the UCLA-GIT 2.0 questionnaire on the same day of US and DUS examination.

### Selection criteria

Consecutively recruited patients with SSc who consented to the assessment of the SMA and/or the IMA flow with DUS.

All included patients were evaluated in the Internal Medicine Ultrasound Clinic of the Azienda Ospedaliero Universitaria Careggi from September 2022 to November 2023 where they underwent a complete abdominal US and DUS evaluation of SMA and IMA. Patients with coronary or peripheral artery disease were excluded.

The study protocol was approved by local Ethical Committee (approval number: 23805_oss).

### Approach to ultrasound

Ultrasound examination was performed after a fasting of at least 8 h by a single operator blinded to clinical features. DUS study was conducted with an Esaote MyLab X8eXP using a 1–8 MHz Convex probe. The probe’s position was adjusted to reach an angle of less than 60°, and the sample volume was regulated to include the vessels’ inner diameter. Every measurement was the mean of three consecutive determinations. For both SMA and IMA, the following parameters were measured: caliber, PSV, EDV, reverse velocity (RV), mean velocity (MV), RI and PI.

### Statistical analyses

Heterogeneity was assessed by *I*^2^ test, whereas Funnel plots were used to detect publication bias for principal endpoints with at least ten trials.

If data from more than one study on a given outcome were available, a meta-analysis using a random-effects model as the primary analysis was performed. Weighted mean differences (WMD) and 95% CIs were calculated for continuous variables. All analyses were performed using Comprehensive Meta Analysis v. 4.

Data were expressed as mean ± standard deviation (Std.dev), or as median (25th–75th percentile), depending on their distribution. Kruskal–Wallis test was used to assess the significance of differences for variables among SSc subsets (sine scleroderma, lcSSc, dcSSc). Linear regression analyses were used to assess the associations between the US parameters (SMA/IMA RI, PI) and continuous variables. Mann-–Whitney test was used to test association among dichotomous variables and SMA/IMA RI, PI values. Multiple regression models were constructed for each parameter, adjusting for potential confounders, including sex, age, and variables that were considered to influence US parameters per the literature review.

All statistical analyses were performed using SPSS 20 (IBM, Armonk, New York, U.S.A.).

A p value < 0.05 was considered statistically significant.

## Results

### Meta-analysis

**Fig. 1:**
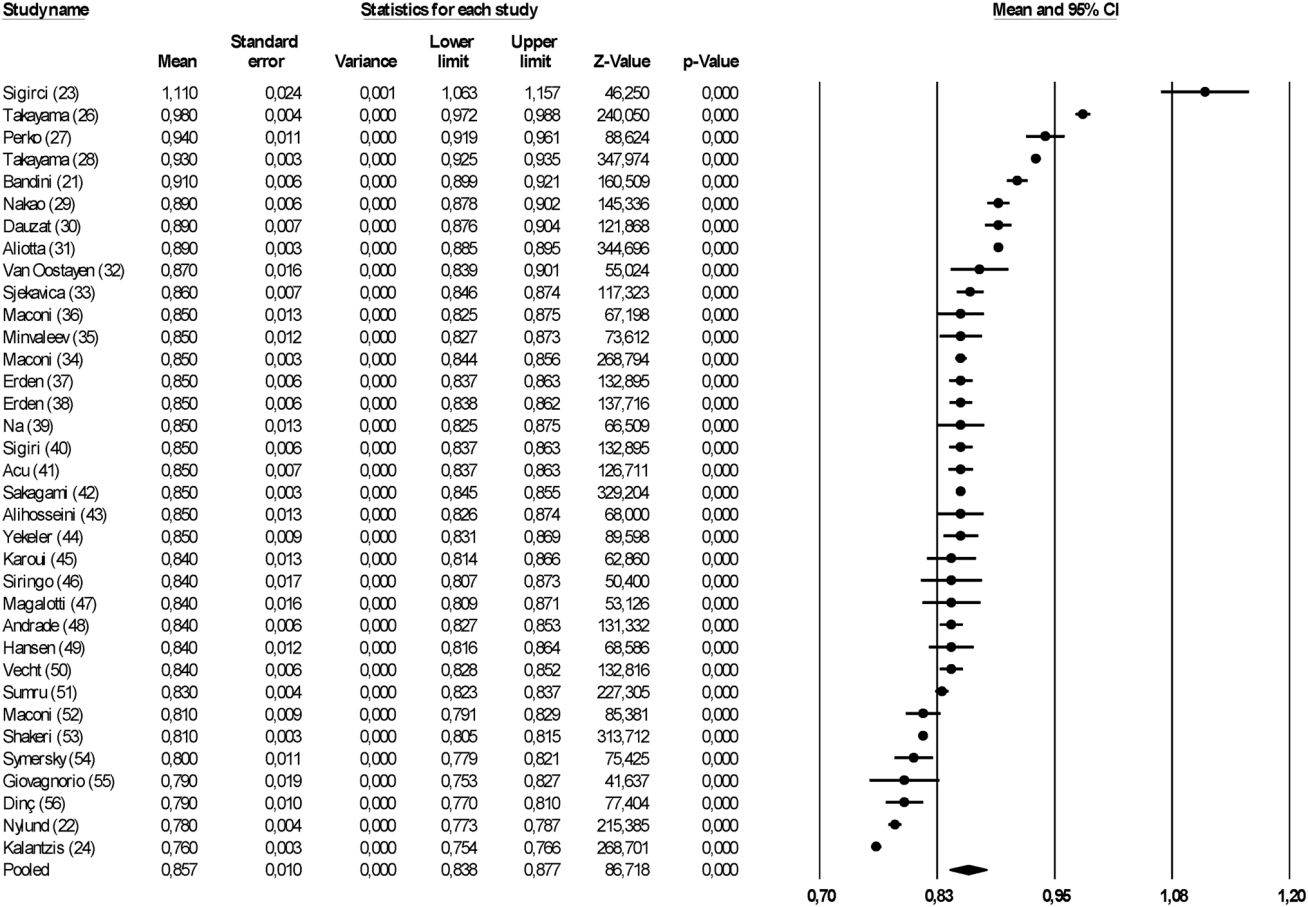
Weighted median value of SMA RI among studies enrolling healthy subjects

The study flow summary is reported in Fig. 1S of Supplementary Materials. The search of Medline and Embase databases allowed the identification of 11,172 items; after deduplication and after excluding studies by reading the title (*n* = 7806), a further 55 studies were excluded after reviewing full-text and reasons for exclusion are reported in Fig. 1S.

### Healthy subjects

Out of 82 studies enrolling 1749 HS and receiving an assessment of mesenteric arterial blood flow, 45 studies reported either SMA or IMA evaluation (Table 1). Out of 45 studies, 35, 23, 3, and 6 reported data on SMA RI, SMA PI, IMA RI, and IMA PI, respectively.

**Table 1:** Main characteristics and DUS parameters of healthy subjects (HS) included studies

First author	Study design	Number of HS included	Mean age (years)	Women (%)	SMA RI	SMA PI	IMA RI	IMA PI
Siğirci 2003 [23]	CS	25	37	60	1.11 ± 0.12	4.24 ± 1.48	1.09 ± 0.07	4.65 ± 1.03
Takayama 2009 [26]	CS	24	31	0	0.98 ± 0.02	4.29 ± 0.4	NR	NR
Perko 1997 [27]	P	8	25	38	0.94 ± 0.03	NR	NR	NR
Takayama 2009 [28]	P	14	35	0	0.93 ± 0.01	4.17 ± 0.19	NR	NR
Bandini 2022 [21]	R	28	36	64	0.91 ± 0.03	4.53 ± 1.03	0.91 ± 0.03	6.08 ± 1.53
Nakao 2022 [29]	P	24	22	58	0.89 ± 0.05	2.95 ± 0.98	NR	NR
Dauzat 1994 [30]	CS	30	31	37	0.89 ± 0.043	NR	NR	NR
Aliotta 1997 [31]	CS	15	30	67	0.89 ± 0.05	NR	NR	NR
Van Oostayen 1994 [32]	CS	10	53	NR	0.87 ± 0.05	NR	NR	NR
Sjekavica 2007 [33]	CS	67	40	48	0.86 ± 0.06	NR	NR	NR
Maconi 1998 [34]	P	40	34	58	0.85 ± 0.02	NR	NR	NR
Minvaleev 2021 [35]	P	12	46	67	0.85 ± 0.04	NR	NR	NR
Maconi 2015 [36]	P	10	40	80	0.85 ± 0.04	2.75 ± 0.75	NR	NR
Erden 1997 [37]	CS	22	32	46	0.85 ± 0.03	3.35 ± 0.93	NR	NR
Erden 1998 [38]	CS	42	43	NR	0.85 ± 0.04	3.07 ± 0.86	NR	NR
Na 2017 [39]	CS	30	39	47	0.85 ± 0.07	NR	NR	NR
Siğirci 2000 [40]	CS	22	42	55	0.85 ± 0.03	3.35 ± 0.93	0.9 ± 0.06	3.57 ± 0.91
Acu 2018 [41]	CS	20	43	50	0.85 ± 0.03	3.81 ± 1.13	NR	NR
Sakagami 2002 [42]	CS	15	59	33	0.85 ± 0.01	2.70 ± 0.16	NR	NR
Alihosseini 2024 [43]	CC	16	30	69	0.85 ± 0.05	NR	NR	NR
Yekeler 2005 [44]	P	10	37	40	0.85 ± 0.03	NR	NR	NR
Karoui 2009 [45]	P	14	33	57	0.84 ± 0.05	1.45 ± 0.16	NR	NR
Siringo 2001 [46]	P	9	33	30	0.84 ± 0.05	NR	NR	NR
Magalotti 2003 [47]	P	10	37	70	0.84 ± 0.05	NR	NR	NR
Andrade 2013 [48]	CS	22	33	68	0.84 ± 0.04	1.88 ± 0.92	NR	NR
Hansen 2009 [49]	CS	6	47	50	0.84 ± 0.06	NR	NR	NR
Vecht 1998 [50]	CS	10	52	20	0.84 ± 0.02	NR	NR	NR
Sumru 2006 [51]	CS	30	43	0	0.83 ± 0.02	2.44 ± 0.13	NR	NR
Maconi 1996 [52]	CS	10	31	60	0.81 ± 0.03	NR	NR	NR
Shakeri 2015 [53]	CC	60	36	50	0.81 ± 0.02	2.20 ± 0.27	NR	NR
Symersky 2007 [54]	P	8	28	62	0.80 ± 0.03	NR	NR	NR
Giovagnorio 1998 [55]	CS	10	28	70	0.79 ± 0.06	NR	NR	NR
Dinç 1998 [56]	CS	24	44	17	0.79 ± 0.05	NR	NR	NR
Nylund 2022 [22]	P	122	48	50	0.78 ± 0.04	NR	NR	NR
Kalantzis 2002 [24]	P	50	37	58	0.76 ± 0.02	NR	NR	NR
Voet 1995 [57]	CS	24	40	NR	NR	6.50 ± 1.30	NR	NR
Sabbà 1991 [58]	P	12	66	0	NR	4.83 ± 0.17	NR	NR
Quarto di Palo 2002 [19]	P	25	29	100	NR	4.13 ± 0.97	NR	NR
Ludwig 1999 [59]	P	13	27	86	NR	3.60 ± 0.90	NR	4.60 ± 0.90
Ludwig 1999 [60]	P	20	31	50	NR	3.40 ± 0.60	NR	4.60 ± 0.90
Iwao 1998 [61]	P	24	62	29	NR	3.07 ± 0.08	NR	NR
Schiedermaier 1999 [62]	P	10	53	40	NR	2.84 ± 0.14	NR	NR
Arienti 1996 [63]	P	9	29	78	NR	2.62 ± 0.13	NR	NR

*CS* cross sectional, *P* prospective, *R* retrospective, *CC* case–control, *NR* not reported

No formal analyses were performed for IMA RI and IMA PI due to the paucity of studies reporting this information.

### SMA RI

**Fig. 2:**
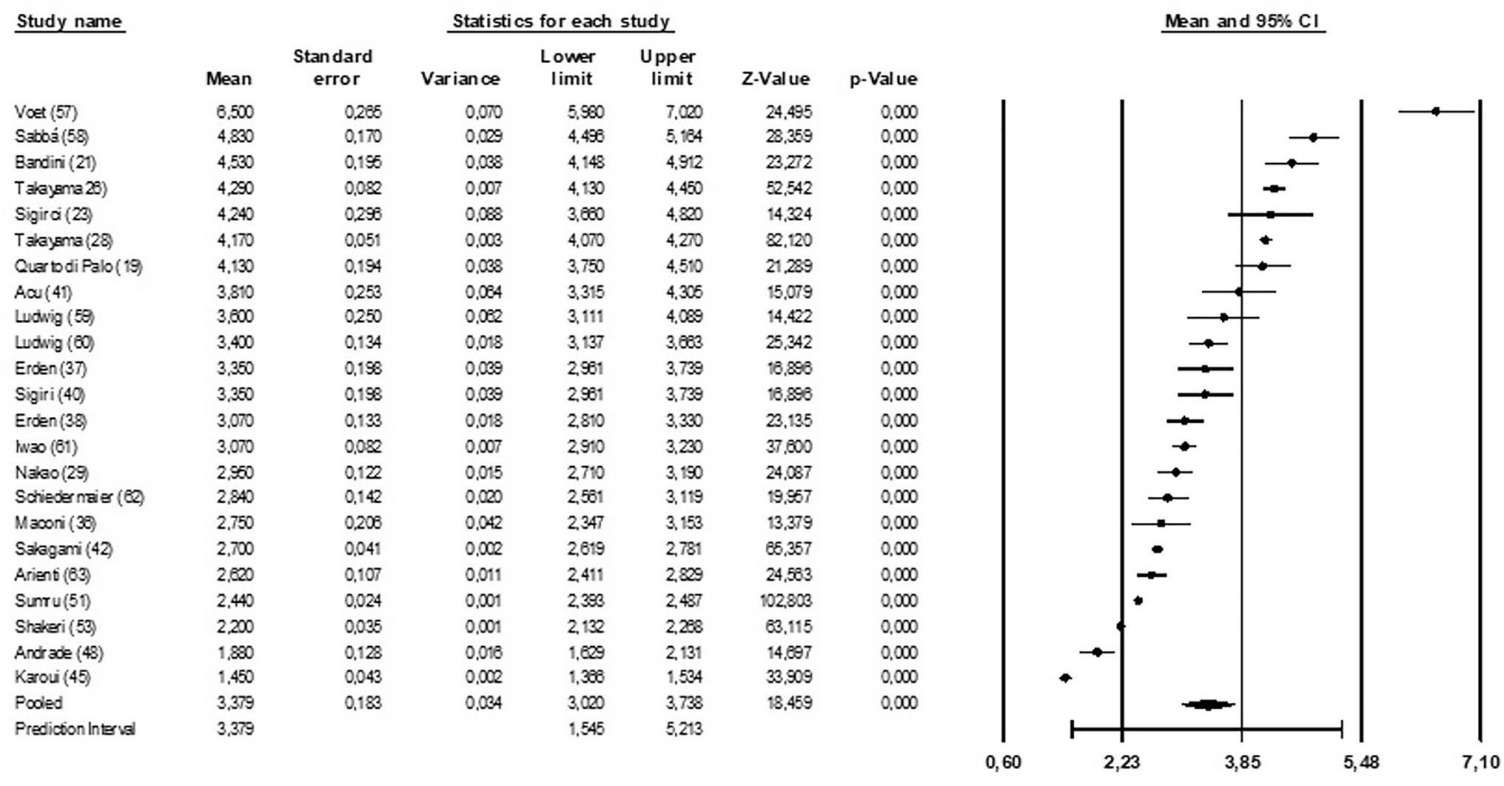
Weighted median value of SMA PI among studies enrolling healthy subjects

Among 45 studies reporting this information and enrolling 869 subjects, the weighted median value of SMA RI was 0.86 [0.84; 0.88], *p* < 0.001 with high heterogeneity (*I*^2^: 99.2%, *p* < 0.001; Fig. 1). After removing the two studies reporting the highest (Siğirci [23]) and lowest (Kalantzis [24]) value of SMA RI, the median value was 0.85 [0.84; 0.87], *p* < 0.001. A visual analysis of funnel plot seems to exclude any possible publication bias (Fig. 2S). To explore possible interactions between SMA RI values and the proportion of women enrolled and mean age at entry, we perform a meta-regression analysis. A significant inverse correlation (slope: − 0.001; intercept 0.91, *p* < 0.001) was found between SMA RI and the proportion of women enrolled (i.e., studies enrolling a higher proportion of women reported lower values of SMA RI). A similar figure was observed for mean age (slope: − 0.002; intercept 0.92, *p* < 0.001) with a significant inverse correlation, meaning that studies enrolling older subjects reported lower values of SMA RI.

### SMA PI

Twenty-three studies, enrolling 517 patients, reported this information. The weighted median value of SMA-PI was 3.38 [3.02; 3.74], *p* < 0.001 with high heterogeneity (*I*^2^:99.3%, *p* < 0.001; Fig. 2). A visual analysis of funnel plot suggests possible publication bias for SMA PI (Fig. 3S). A significant inverse correlation (slope: − 0.03; intercept 3.91, *p* < 0.001) was found between SMA PI and the proportion of women enrolled (i.e., studies enrolling a higher proportion of women reported lower values of SMA PI). A direct correlation between age and SMA PI was observed (slope: 0.005; intercept 2.42, *p* < 0.001), meaning that studies enrolling older subjects reported higher values of SMA PI.

### Ssc patients

Four studies, including the present one, reported SMA DUS evaluation. Two studies, including the present one, reported IMA DUS evaluation (Table 2).

**Table 2:** Main characteristics and DUS parameters of SSc patients included studies

First author	#SSc	#Control	Age (years)	Women (%)	Disease duration (years)	SMA RI	SMA PI	IMA RI	IMA PI
Quarto di Palo 2002 [19]	27	25	51 ± 12	100	7.3 ± 5.9	NR	3.51 ± 0.95 (lcSSc) 3.48 ± 1.12 (dcSSc)	NR	NR
Salera 2005 [20]	50	–	44.5 ± 14.5	70	NR	0.78 ± 0,009	NR	NR	NR
Bandini 2022 [21]	28	28	49 ± 12	89.3	7 (2–9)	0.88 ± 0.04	3.33 ± 0.75	0.88 ± 0.04	3.54 ± 0.95
Bandini 2024	78	–	57.2 ± 13.1	87.2	9.9 ± 7.1	0.86 ± 0.04	2.85 ± 0.70	0.86 ± 0.05	3.19 ± 0.66

Among the four studies reporting SMA evaluation and enrolling 183 patients, three reported information about SMA RI. The weighted median value of SMA RI was 0.84 [0.77; 0.91], *p* < 0.003 with high heterogeneity (*I*^2^:86.67%, *p* < 0.002).

Similarly, among the four studies reporting SMA evaluation and enrolling 183 patients, three reported information about SMA PI. The weighted median value of SMA PI was 3.20 [2.78; 3.62], *p* < 0.001 with high heterogeneity (*I*^2^: 86.8%, *p* < 0.001).

No formal analyses were performed for IMA RI and IMA PI due to the paucity of studies reporting this information.

## Cohort study

### Characteristics of the cohort

Among 78 enrolled patients, 30 (39%) were classified as diffuse SSc (dcSSC), 35 (45%) as limited cutaneous SSc (lcSSc) and 13 (17%) as sine scleroderma (Table 3). Patients’ age, sex, and disease duration were similar across groups (*p* > 0.05). As expected, patients with diffuse cutaneous disease had significantly higher skin scores and worse pulmonary function as measured by FVC and DLCO (*p* < 0.05). Interestingly, patients in the sine scleroderma group had more significant GI symptoms as assessed by the total score on the UCLA GIT 2.0 survey (particularly driven by reflux and abdominal distention) than the other two groups. Immunosuppressive therapies, including rituximab and cyclophosphamide, were utilized significantly more among patients with diffuse disease than in the other groups (*p* < 0.05). Otherwise, there were significant differences across groups in the utilization of proton pump inhibitors (PPI’s) and pentoxifylline.

**Table 3:** Patients’ characteristics and their differences between dcSSc, lcSSc and sine scleroderma

	All (*n* = 78)	Sine scleroderma (*n* = 13)	lcSSc (*n* = 35)	dcSSc (*n* = 30)	*p* for trend
Age^§^ (*n* = 78, 100%)	57.5 ± 13.1	58 ± 11.6	60 ± 11	52.9 ± 15.3	0.161
Sex F^#^ (*n* = 78, 100%)	68 (87.2)	13 (100)	31 (68.1)	24 (82.8)	0.293
Disease duration**^** (*n* = 78, 100%)	8 (5*–*13)	10 (6*–*12.50)	8 (6–15.75)	6 (3–12.50)	0.537
ACA^**#**^ (*n* = 76, 97.4%)	29 (38.2)	5 (38.5)	24 (66.7)	0 (0)	0.000*
Scl70^#^ (*n* = 76, 97.4%)	34 (44.7)	4 (30.8)	8 (22.2)	22 (81.5)	0.000*
mRSS^ (*n* = 55, 70.5%)	3 (0*–*7)	0 (0*–*0)	2.50 (0*–*4.00)	10 (6*–*16.50)	0.000*
ESR^ (*n* = 53, 67.9%)	17 (10*–*33)	2 (3*–*37)	14 (10*–*27)	24 (13*–*43.5)	0.237
CRP^ (*n* = 52, 66.7%)	0.21 (0.12*–*0.30)	0.21 (0.04*–*0.33)	0.20 (0.02*–*0.30)	0.26 (0*–*0.36)	0.819
FVC^ (*n* = 51, 65.4%)	101 (86*–*110)	106 (93*–*113)	102 (95.5*–*109.75)	76 (68.5*–*96.5)	0.001*
FEV1^ (*n* = 45, 57.7%)	96 (78*–*105)	104 (91*–*115)	96.5 (83.5*–*102.75)	77 (67.5*–*101)	0.081
DLCO^ (*n* = 52, 66.7%)	72 (56.2*–*72.0)	72.5 (61*–*80.75)	77 (66.25*–*87.50)	57 (38.25*–*78.5)	0.028*
GIT 2.0 reflux^ (*n* = 74, 94.9%)	0.5 (0.25*–*0.78)	0.87 (0.19*–*1.50)	0.30 (0.12*–*0.65)	0.50 (0.25*–*0.75)	0.066
GIT 2.0 abdominal distension**^** (*n* = 74, 94.9%)	0.75 (0.25*–*1.50)	1.50 (0.75*–*2.5)	0.75 (0.19*–*1.56)	0.75 (0.25*–*1.50)	0.065
GIT 2.0 fecal incontinence**^** (*n* = 74, 94.9%)	0 (0*–*0.25)	0 (0*–*0.25)	0 (0*–*0)	0 (0*–*0)	0.777
GIT 2.0 diarrhea**^** (*n* = 74, 94.9%)	0 (0*–*0.50)	0.5 (0*–*0.75)	0 (0*–*0.5)	0 (0*–*0.5)	0.215
GIT 2.0 social activities**^** (*n* = 74, 94.9%)	0.16 (0*–*0.50)	0.16 (0*–*0.50)	0.16 (0*–*0.50)	0.16 (0*–*0.50)	0.994
GIT 2.0 emotional wellbeing**^** (*n* = 73, 93.6%)	0 (0*–*0.33)	0.22 (0*–*0.50)	0 (0*–*0.39)	0 (0*–*0.33)	0.333
GIT 2.0 total score**^** (*n* = 78, 100%)	0.33 (0.14*–*0.62)	0.51 (0.33*–*0.84)	0.24 (0.08*–*0.52)	0.33 (0.19*–*0.54)	0.039*
GIT 2.0 constipation**^** (*n* = 78, 100%)	0.25 (0*–*0.75)	0.25 (0*–*0.75)	0.25 (0*–*0.75)	0 (0*–*0.75)	0.819
EF**^** (*n* = 49, 62.8%)	62 (60*–*67.50)	65 (61.8*–*70)	62.5 (60*–*67.5)	60 (60*–*65)	0.106
PAPs**^** (*n* = 51, 65.4%)	25 (21*–*30)	25 (20*–*35)	25 (21*–*29)	25.5 (21.5*–*31.75)	0.656
Creatinine clearance* (*n* = 39, 50%)	90 (70*–*104)	88 (66*–*97.5)	93.65 (72.75*–*104.75)	83 (69.5*–*151.5)	0.798
RRI right**^** (*n* = 45, 57.7%)	0.66 (0.63*–*0.71)	0.71 (0.68*–*0.75)	0.65 (0.63*–*0.71)	0.65 (0.59*–*0.69)	0.019*
RRI left^ (*n* = 45, 57.7%)	0.67 (0.64*–*0.71)	0.71 (0.67*–*0.76)	0.67 (0.64*–*0.70)	0.65 (0.60*–*0.71)	0.028*
Digital ulcers^#^ (*n* = 64,82.1%)	40 (5.3)	6 (46.2)	17 (47.2)	17 (58.6)	0.045*
NVC pattern^**#**^					
Not-specific	4 (5.1)	2 (15.4)	1 (2.8)	1 (3.4)	0.366
Early	15 (19.2)	3 (23.1)	9 (25)	3 (10.3)	
Active	21 (29.9)	3 (23,1)	13 (36.1)	5 (17.2)	
Late (*n* = 44, 56.4%)	4 (5.1)	1 (7.7)	0 (0)	3 (10.3)	
ILD^#^	32 (58.2)	5 (9.1)	6 (10.9)	21 (38.2)	0.050
(*n* = 55, 70.5%)					
HCQ^#^ (*n* = 59, 75.6%)	32 (41.0)	6 (46.2)	20 (55.6)	6 (20.7)	0.084
MMF^#^ (*n* = 64, 82.1%)	37 (47.4)	6 (46.2)	14 (38.9)	17 (58.6)	0.033
MTX^#^ (*n* = 59, 75.6%)	2 (2.6)	0 (0)	2 (5.6)	0 (0)	0.374
AZA^#^ (*n* = 59, 75.6%)	4 (5.1)	1 (7.7)	0 (0)	1 (7.7)	0.083
TCZ^#^ (*n* = 59, 75.6%)	1 (1.3)	0 (0)	0 (0)	1 (3.4)	0.320
RTX^#^ (*n* = 60, 76.9%)	6 (7.7)	1 (7.7)	5 (16.7)	5 (17.2)	0.009*
CYC^#^ (*n* = 59, 75.6%)	8 (10.3)	1 (7.7)	1 (2.8)	6 (20.7)	0.013*
IVIg^#^ (*n* = 78, 100%)	0 (0)	0 (0)	0 (0)	0 (0)	1.000
Calcium antagonists^#^ (*n* = 62, 79.5%)	23 (29.5)	4 (30.8)	13 (36.1)	6 (20.7)	0.610
Pentoxifylline^#^ (*n* = 59, 75.6%)	12 (15.4)	5 (38.5)	6 (16.7)	1 (3.4)	0.037*
Iloprost^#^ (*n* = 59, 75.6%)	18 (23.1)	2 (15.4)	9 (25.0)	11 (37.9)	0.505
PGE^#^ (*n* = 59, 75.6%)	2 (2.6)	2 (15.4)	2 (5.6)	0 (0)	0.374
Sildenafil^#^ (*n* = 59, 75.6%)	20 (25.6)	3 (23.1)	10 (27.8)	7 (24.1)	0.814
Bosentan^#^ (*n* = 60, 76.9%)	33 (42.3)	7 (53.8)	14 (38.9)	12 (41.4)	0.437
Riociguat^#^ (*n* = 59, 75.6%)	0 (0)	0 (0)	0 (0)	0 (0)	1.000
Macitentan^#^ (*n* = 59, 75.6%)	0 (0)	0 (0)	0 (0)	0 (0)	1.000
PPI^#^ (*n* = 71, 91.0%)	60 (76.9)	1 (7.7)	31 (86.1)	22 (75.9)	0.023*
SMA RI^ (*n* = 77; 98.7%)	0*.*87 (0*.*84–0*.*89)	0*.*84 (0*.*81–0*.*89)	0*.*87 (0*.*83–0*.*88)	0.88 (0.83–0.90)	0.032*
SMA PI^ (*n* = 77; 98.7%)	2*.*82 (2*.*35–3*.*31)	2*.*4 (1*.*9–2*.*7)	2*.*7 (2*.*2–3*.*0)	3.1 (2.8–3.7)	0.004*
IMA RI^ (*n* = 66; 84.6%)	0*.*86 (0*.*84–0*.*89)	0*.*84 (0.79–0.87)	0*.*89 (0*.*85–0*.*9)	0.86 (0.85–0.89)	0.10
IMA PI^ (*n* = 66; 84.6%)	3*.*21 (2*.*72–3*.*66)	2*.*83 (2*.*37–3*.*5)	3*.*33 (2*.*88–3*.*81)	3*.*21 (2*.*63–3*.*43)	0.27

^*^p < 0.05

^§^mean ± dev. standard

^median (interquartile range 25^th^–75th)

^#^*n* (%)

*lcSSC* limited cutaneous systemic sclerosis, *dcSSc* diffuse cutaneous systemic sclerosis, *ESR* erithrocyte sedimentation rate, *CRP* C-reactive protein, *FVC* forced vital capacity, *FEV*1 forced expiratory volume in the first 1st second, *DLCO* diffusion lung CO, *FE* (left ventricular) ejection fraction, *PAPs* pulmonary arterial pressures, *SMA* superior mesenteric artery, IMA inferior mesenteric artery, *RI* resistive index, *PI* pulsatility index, *RRI* renal resistive index, *NVC* nailfold videocapillaroscopy, *mRSS* modified Rodnan skin score

### Differences in the mesenteric vasculature detected by DUS in subgroups of ssc and associate with distinct GI and extraintestinal features

#### SMA

The median value of SMA RI and PI were 0.86 ± 0.04 and 2.85 ± 0.70 in the entire population.

A significant between-group difference for PI (*p* for trend = 0.004) and RI (*p* for trend = 0.032) was observed for patients with different SSc subsets; patients with sine scleroderma and lcSSc showed lower SMA RI and PI median values (0.84 and 0.87, and 2.4 and 2.7, respectively) in comparison with dcSSc (0.88 and 3.1) (Table 3). Median SMA PI values were significantly lower in females than males (2.73 *vs* 3.39, *p* = 0.004;). Higher SMA RI and PI values were observed in patients with Scl70 autoantibodies (0.88 vs 0.85, *p* = 0.006 and 3.05 vs 2.65, *p* = 0.030, respectively). SMA RI values were higher in patients with DUs (0.87 vs 0.85, *p* = 0.026). Lower SMA RI median values were observed in patients with ACA autoantibodies (0.85 vs 0.87, *p* = 0.015), and in patients with diarrhea (0.85 vs 0.87, *p* = 0.078). Both SMA RI and PI were higher in patients treated with bosentan (0.88 vs 0.85, *p* = 0.008 and 3.08 vs 2.6, *p* = 0.009 respectively) (Table S2).

We then sought to determine whether DUS measures also correlated with the severity of SSc clinical features. SMA PI and RI were both directly correlated with mRSS (*ῥ*: 0.39, *p* = 0.030 and *ῥ* 0.44, *p* = 0.001, respectively) and inversely correlated with the index of pulmonary function FVC (*ῥ* − 0.39, *p* = 0.004 and *ῥ* − 0.28, *p* = 0.044). A lower SMA PI correlated with higher PAPs (*ῥ* − 0.32, *p* = 0.020), and right and left RRI (*ῥ* − 0.34, *p* = 0.023) (Table S3).

Though we were able to identify differences between subgroups of patients with SSc, when comparing SMA RI obtained in patients with SSc (0.84 ± 0.43) with that of HS (i.e., a meta-analysis reported above: 0.85 ± 0.30), no between-group difference was observed (*p* = 0.72). The corresponding figure for SMA-PI was 3.20 ± 2.77 for SSc and 3.38 ± 4.16 for HS with an insignificant between-group difference (*p* = 0.64).

#### IMA

The median value of IMA RI and PI were 0.86 ± 0.05 and 3.19 ± 0.66 in the entire population. Median IMA RI values were significantly lower in patients with diarrhea (0.85 vs 0.88, *p* = 0.026). No other significant differences were observed among different subgroups of patients (Table S4).

Both IMA RI and PI were directly correlated with age (*ῥ* 0.32, *p* = 0.011 and *ῥ* 0.27, *p* = 0.042, respectively) and inversely correlated with UCLA GIT 2.0 incontinence score (*ῥ* − 0.33, *p* = 0.008 and *ῥ* − 0.30, *p* = 0.021, respectively) (Table S3).

All the other insignificant correlations for both SMA and IMA are reported in Supplementary Materials (Table S5 and S6).

### Significant associations between vascular findings on DUS and ssc features remaining after adjusting for potential confounders

We performed multivariate linear regression analyses, adjusting for age and sex, and including all covariates reaching a statistical significance at univariate analyses (Table S2, S3, and S4).

In the adjusted model, SMA RI remained significantly directly correlated with the presence of anti-Scl70 antibodies (*β* 0.33; *p* = 0.004), DUs (β 0.26; *p* = 0.038), and higher mRSS values (*β* 0.37; *p* = 0.012) (Table S7 and S8). A significant inverse correlation was confirmed between SMA RI and ACA (*β* − 0.32; *p* = 0.006). When looking at SMA PI, a persistent statistically significant direct correlation was observed with anti-Scl70 antibodies (*β* 0.23; *p* = 0.047), higher mRSS values (*β* 0.42; *p* = 0.002), and treatment with bosentan (*β* 0.28; *p* = 0.027). A significant inverse correlation was observed with FVC (*β* − 0.37; *p* = 0.007), and PAPs (*β* − 0.31; *p* = 0.024).

When looking at associations with IMA, an inverse correlation was confirmed both for IMA RI and PI with higher UCLA-GIT 2.0 incontinence scores (*β* − 0.32, *p* = 0.012 and *β* − 0.30, *p* = 0.020, respectively).

A multivariate linear regression analysis including covariates showing a significant correlation after adjusting for age and sex (mRSS, ACA, Bosentan therapy; Table S7), persistent significant direct correlations with both SMA RI and PI were observed for mRSS (*β* 0.248, *p* = 0.030 and *β* 2.995, *p* = 0.004, respectively) and Bosentan (*β* 0.400, *p* = 0.003 and *β* 3.508, *p* = 0.001, respectively), but not for ACA (Table S8).

## Discussion

Our data clearly show that DUS can be helpful in evaluating the splanchnic district of SSc patients. To date, only a few studies have investigated the mesenteric circulation, that is the GI macrovascular involvement, in SSc patients [19–21].

In 2002 Quarto di Palo et al. [19] conducted a preliminary DUS study of the SMA describing changes in basal SMA flow, even in patients without GI symptoms. In 2022 we then confirmed that hemodynamics alterations of the splanchnic vessels are noninvasively detectable with DUS in patients with SSc [21]. In both these preliminary studies the correlation with other organ involvement could not be assessed given of the small number of patients enrolled.

In the present study, the splanchnic circulation was evaluated in a larger cohort of 78 SSc patients, and only in one case it was impossible to evaluate SMA blood flow given the intense meteorism. Instead, DUS study of the IMA was possible in 66 out of 78 patients confirming that, even though IMA is a very small vessel and patients with SSc often complaints of abdominal bloating, the artery can be noninvasively examined. Therefore, DUS appears to be confirmed as a non-invasive, repeatable and patient-friendly technique very useful in achieving information about the whole GI vasculature of patients with SSc.

To date, there are no objective markers or tests that can help clinician quantifying the vascular damage or to assess vascular disease activity and progression, especially for what concerns GI involvement. In this perspective, the possibility to evaluate the splanchnic vasculature with DUS could be of great importance both for the definition of GI vascular damage but even in the follow up and possibly to evaluate response to therapies.

A strength of the present study is that it was conducted on a significant number of SSc patients, and therefore we obtained greater statistical power than in our previous pilot study. This allowed us to evaluate possible correlations between the main semiquantitative DUS parameters (i.e., RI and PI) and disease characteristics as well as other parameters of organ involvement.

Interestingly, both SMA RI and SMA PI were significantly lower in patients with sine scleroderma and lcSSc compared with dcSSc patients. This may suggest that vasculopathy of the splanchnic district may vary with the different clinical subtype. It has already been hypothesized that sine cutaneous scleroderma and lcSSc may share demographic, clinical, and immunologic features [25], and therefore our results may support this hypothesis for gastrointestinal involvement, including significant vascular alterations in disease pathogenesis, as well.

Regarding the correlation between DUS hemodynamic values and the demographic, clinical, and bio-humoral parameters of our patient cohort, another very interesting result is the statistically significant correlation between the RI and PI values of the IMA and specific symptoms of UCLA-GIT 2.0 questionnaire such as diarrhea and fecal incontinence. In fact, there are very few data in the extant literature concerning the large-bowel vasculature, which is almost exclusively deputed to the IMA, due to of its small diameter and anatomic course. In the present study not only the possibility to study the fasting blood flow of the inferior mesenteric artery in patients with SSc was confirmed, but DUS data showed a possible correlation between IMA splanchnic vascular dysfunction and symptoms related to large-bowel involvement. Since lower GI-tract involvement is responsible for some of the most troublesome symptoms observed in patients with SSc, significantly affecting even their social activities, the diagnosis of lower GI involvement is of great importance. However, this is currently very challenging and often supported by invasive and/or expensive investigations. Therefore, the possibility to noninvasively study the principal artery involved in the perfusion of the lower gastrointestinal tract, could open up new diagnostic opportunities, especially in the early asymptomatic or paucisymptomatic stages of the disease.

Our multivariate statistical analysis confirmed a significant correlation between the higher values of SMA RI and SMA PI with bosentan therapy, but not with all other (including vascular-acting) drug therapies analyzed. Although these should be considered as preliminary data, it would be very interesting to hypothesize a possible specific class effect of endothelial receptor antagonists, drugs characterized by very potent vasoactive (e.g., endothelial protective) activity.

Our review of the literature confirmed that only a few studies have evaluated the SMA and IMA blood flow of SSc patients with DUS. Regarding HS, these studies are much more numerous, especially concerning the SMA. However, significant heterogeneity is observed among the included studies both for SMA RI and SMA PI values, and with a possible publication bias for the latter one. Our meta-analysis showed that both SMA RI and SMA PI have a significant correlation with both age and sex. Differences between weighted median values of SMA RI of HS and SSc patients were not significant, nor were the difference between weighted median values of SMA PI of HS and SSc patients. These data should be carefully interpreted since there are significant differences in the sample sizes of the included HS and SSc patients, and due to the great heterogeneity of HS and SSc studies included.

What clearly emerges from the review of the literature is that the normal values of semi-quantitative Doppler parameters of the SMA (with particular reference in this case to the pulsatility index) and even more so of the inferior mesenteric artery are currently not certain. Furthermore, there are no reliable data concerning possible variations of these parameters with age, sex, or BMI. Further studies are needed to find out what the values of the principal DUS parameters of HS are (i.e., not only RI and PI) and how much they are affected by variables such as age, sex, and BMI.

The present study has some limitations that should be considered in the interpretation of the findings. Although we studied a significant size (*n* = 78) of patients with SSc (especially when considering the rarity of the disease), the sample is still not large enough to draw definitive conclusions. Even though significant efforts were made to adjust for potentially confounding factors using multivariate analysis, larger sample size is needed to confirm these preliminary data.

Moreover, a single operator performed all DUS evaluations. Despite being a trained operator, this could have limited the accuracy of our data, and future studies could examine inter-rater differences.

Finally, a control group of HS matched for sex, age and anthropometric characteristics would be necessary to compare DUS data.

Further studies are necessary to confirm these preliminary correlations found between vascular GI and other organs/clinical involvement and to further explore the relationship between the GI vasculature and other signs, symptoms and objective evidence of GI involvement in SSc. Moreover, prospective data are mandatory to determine if DUS can play a role in predicting SSc GI-disease progression.

## Conclusions

Our study confirms that splanchnic vascular involvement can be noninvasively evaluated in patients with SSc by DUS, and provides novel insight into GI vascular involvement and tentatively with relevant disease manifestations. DUS may provide a novel window into the complex disease pathogenesis of SSc, including opportunities to early (and even subclinical) detect GI vascular involvement, and as a potential imaging marker for the assessment of drug efficacy. To advance this exciting field, further studies are necessary both to confirm these preliminary observations, and to obtain definitive data regarding normal (i.e., healthy subjects) and SSc patients SMA and IMA fasting DUS parameters.

## Supplementary information

Below is the link to the electronic supplementary material.

Supplementary file1 (DOCX 185 KB)

## Data Availability

Data are available, upon reasonable request, contacting the corresponding author.
