# Supplementary material for: Doppler ultrasound, a noninvasive tool for the study of mesenteric arterial flow in systemic sclerosis: a cross-sectional study of a patient cohort with review and meta-analysis of the literature
Source: Intern Emerg Med. 2024 Oct 16;20(2):381–94. doi: 10.1007/s11739-024-03783-5 (PMC11950030; doi:10.1007/s11739-024-03783-5)
Supplement: Supplementary file 1 — Supplementary file1 (DOCX 185 KB) [file 11739_2024_3783_MOESM1_ESM.docx]

**SUPPLEMENTARY MATERIALS**

**Figure 1S –** Study flow summary


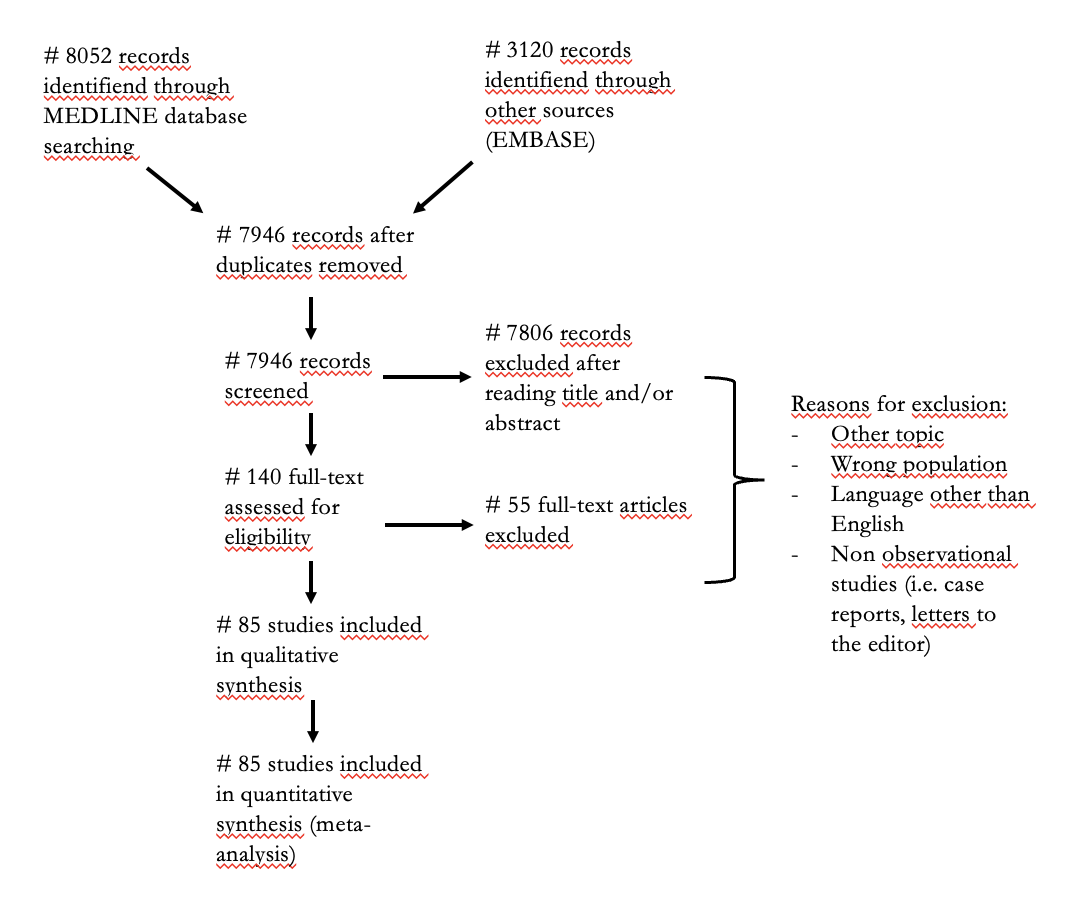


**Figure 2S –** Funnel plot for SMA RI for studies enrolling healthy subjects.

**Figure 3S –** Funnel plot for SMA PI for studies enrolling healthy subjects.

**TABLES**

**Table S1.** Detailed information on search string strategy

| **Limits:** Observational studies; any date up to February 1^st^, 2024 |
| --- |
| **MEDLINE** (N= 8,055 items)  ***Search***: mesenteric artery AND ultrasound  ("mesenteric arteries"[MeSH Terms] OR ("mesenteric"[All Fields] AND "arteries"[All Fields]) OR "mesenteric arteries"[All Fields] OR ("mesenteric"[All Fields] AND "artery"[All Fields]) OR "mesenteric artery"[All Fields]) AND ("diagnostic imaging"[MeSH Subheading] OR ("diagnostic"[All Fields] AND "imaging"[All Fields]) OR "diagnostic imaging"[All Fields] OR "ultrasound"[All Fields] OR "ultrasonography"[MeSH Terms] OR "ultrasonography"[All Fields] OR "ultrasonics"[MeSH Terms] OR "ultrasonics"[All Fields] OR "ultrasounds"[All Fields] OR "ultrasound s"[All Fields])  Translations  mesenteric artery: "mesenteric arteries"[MeSH Terms] OR ("mesenteric"[All Fields] AND "arteries"[All Fields]) OR "mesenteric arteries"[All Fields] OR ("mesenteric"[All Fields] AND "artery"[All Fields]) OR "mesenteric artery"[All Fields]  ultrasound: "diagnostic imaging"[Subheading] OR ("diagnostic"[All Fields] AND "imaging"[All Fields]) OR "diagnostic imaging"[All Fields] OR "ultrasound"[All Fields] OR "ultrasonography"[MeSH Terms] OR "ultrasonography"[All Fields] OR "ultrasonics"[MeSH Terms] OR "ultrasonics"[All Fields] OR "ultrasounds"[All Fields] OR "ultrasound's"[All Fields] |
| **EMBASE** (n= 3,120)  ('mesenteric artery'/exp OR 'mesenteric artery' OR (mesenteric AND ('artery'/exp OR artery))) AND ('ultrasound'/exp OR 'ultrasound' OR (('ultrasound'/exp OR ultrasound) AND ('ultrasound'/exp OR ultrasound))) AND [embase]/lim NOT ([embase]/lim AND [medline]/lim) |
| **Additional search:**  Additional manual search of the references of included studies and former meta-analyses was carried out to identify other newly published and unpublished studies. Completed but yet unpublished studies with the procedures specified above were searched in the www.clinicaltrials.gov register using the same search string as above. |

**Table S2. SMA RI and SMA PI values in different subgroup of patients (only statistically significant differences were reported) in subgroup of patients**

|  | SMA RI median values | | p | SMA PI median values | | p |
| --- | --- | --- | --- | --- | --- | --- |
| Sex (F vs M) | 0.86  (0.84 – 0.89) | 0.89  (0.84 – 0.91) | 0.178 | 2.73  (2.32 – 3.18) | 3.39  (2.86 – 4.19) | *0.004** |
| ACA# (yes vs no) | 0.87  (0.85 – 0.89) | 0.85  (0.81 – 0.88) | *0.015** | 2.93  (2.52 – 3.34) | 2.54  (2.13 – 3.04) | 0.070 |
| Scl70# (yes vs no) | 0.85  (0.82 – 0.87) | 0.88  (0.85 – 0.90) | *0.006** | 2.65  (2.21 – 3.03) | 3.05  (2.60 – 3.40) | *0.030** |
| Digital ulcers# (yes vs no) | 0.85  (0.82 – 0.88) | 0.87  (0.84 – 0.90) | *0.026** | 2.62  (2.40 – 3.04) | 2.90  (2.33 – 3.40) | 0.214 |
| Diarrhea# (yes vs no) | 0.87  (0.85 – 0.89) | 0.85  (0.82 – 0.89) | *0.078** | 2.90  (2.50 – 3.33) | 2.72  (2.13 – 3.30) | 0.288 |
| Bosentan# (yes vs no) | 0.85  (0.82 – 0.88) | 0.88  (0.85 – 0.89) | *0.008** | 2.60  (2.21 – 3.01) | 3.08  (2.55 – 3.66) | *0.009** |

# (Mann-Whitney test)

* p significative if <0.05

**Table S3. Significant correlations between continuous variables (Spearman test)**

|  | SMA RI^§^ | | SMA PI^§^ | | IMA RI^§^ | | IMA PI^§^ | |
| --- | --- | --- | --- | --- | --- | --- | --- | --- |
|  | ῥ | p | ῥ | p | ῥ | p | ῥ | p |
| Age | -0.11 | 0.348 | -0.13 | 0.272 | 0.32 | *0.011** | 0.27 | *0.042** |
| mRSS | 0.44 | *0.001** | 0.39 | *0.030** | 0.07 | 0.632 | 0.09 | 0.535 |
| FVC | -0.28 | *0.044** | -0.39 | *0.004** | -0.17 | 0.284 | -0.31 | 0.056 |
| GIT 2.0 incontinence | -0.05 | 0.667 | -0.00 | 0.972 | -0.33 | *0.008** | -0.30 | *0.021** |
| PAPs | -0.16 | 0.281 | -0.32 | *0.020** | 0.25 | 0.119 | 0.09 | 0.599 |
| RRI right | -0.08 | 0.600 | -0.34 | *0.023** | -0.14 | 0.434 | 0.08 | 0.646 |
| RRI left | -0.15 | 0.337 | -0.34 | *0.023** | -0.13 | 0.466 | 0.18 | 0.308 |

§ correlation coefficient

* p significative if <0.05

**Table S4**. **Significant correlations between categorical variables (Mann-Whitney test)**

|  | IMA RI median values | | p | IMA PI median values | | p |
| --- | --- | --- | --- | --- | --- | --- |
| Sex (F vs M) | 0.86  (0.84 – 0.89) | 0.85  (0.85 – 0.90) | 0.716 | 3.21  (2.73 – 3.60) | 3.25  (2.37 – 4.22) | 0.639 |
| ACA# (yes vs no) | 0.86  (0.84 – 0.89) | 0.86  (0.85 – 0.90) | 0.675 | 3.25  ( 2.74 – 3.56) | 3.16  (2.50 – 3.71) | 0.882 |
| Scl70# (yes vs no) | 0.86  (0.85 – 0.90) | 0.86  (0.84 – 0.89) | 0.741 | 3.21  ( 2.70 – 3.70) | 3.16  (2.70 – 3.50) | 0.825 |
| Digital ulcers# (yes vs no) | 0.86  (0.84 – 0.90) | 0.87  (0.85 – 0.90) | 0.633 | 3.40  (2.90 – 3.67) | 3.21  (2.50 – 3.70) | 0.590 |
| Diarrhea# (yes vs no) | 0.88  (0.85 – 0.90) | 0.85  (0.83 – 0.88) | *0.026** | 3.33  (2.92 – 3.67) | 3.07  (2.44 – 3.50) | 0.126 |
| Bosentan# (yes vs no) | 0.85  (0.82 – 0.88) | 0.88  (0.85 – 0.89) | 0.239 | 3.28  (2.90 – 3.67) | 3.33  (2.78 – 3.67) | 0.817 |

# (Mann-Whitney test)

* p significative if <0.05

**Table S5. Non-significant correlations between continuous variables (Spearman test)**

|  | SMA RI | | SMA PI | | IMA RI | | IMA PI | |
| --- | --- | --- | --- | --- | --- | --- | --- | --- |
|  | ῥ | p | ῥ | p | ῥ | p | ῥ | p |
| Disease duration | 0.08 | 0.485 | -0.02 | 0.867 | 0.12 | 0.345 | 0.05 | 0.732 |
| ESR | -0.20 | 0.160 | -0.21 | 0.139 | 0.01 | 0.937 | 0.05 | 0.777 |
| CRP | 0.20 | 0.162 | 0.01 | 0.921 | 0.29 | 0.061 | 0.12 | 0.444 |
| FEV1 | -0.5 | 0.742 | -0.9 | 0.576 | -0.8 | 0.667 | -0.12 | 0.503 |
| DLCO | -0.02 | 0.864 | -0.02 | 0.907 | 0.04 | 0.824 | 0.01 | 0.955 |
| GIT 2.0 reflux | -0.05 | 0.672 | -0.11 | 0.352 | -0.06 | 0.631 | 0.07 | 0.594 |
| GIT 2.0 abdominal distension | 0.00 | 0.998 | -0.09 | 0.412 | -0.19 | 0.149 | -0.22 | 0.098 |
| GIT 2.0 diarrhea | -0.05 | 0.667 | -0.00 | 0.972 | -0.21 | 0.107 | -0.48 | 0.724 |
| GIT 2.0 social activities | -0.17 | 0.154 | -0.13 | 0.266 | -0.19 | 0.138 | -0.19 | 0.146 |
| GIT 2.0 emotional wellbeing | -0,14 | 0,252 | -0,22 | 0,064 | -0,22 | 0,095 | -0,07 | 0,583 |
| GIT 2.0 constipation | 0.09 | 0.414 | -0.11 | 0.377 | 0.13 | 0.335 | 0.01 | 0.951 |
| FE | -0.04 | 0.781 | -0.08 | 0.604 | 0.16 | 0.353 | 0.12 | 0.488 |
| Creatinine clearance | 0.24 | 0.137 | 0.25 | 0.127 | -0.18 | 0.327 | -0.01 | 0.962 |

* p significative if <0.05

**Table S6. Non-significant correlations between categorical variables (Mann-Whitney test)**

|  | SMA RI p | SMA PI p | IMA RI  p | IMA PI  p |
| --- | --- | --- | --- | --- |
| RNA pol III | 0.556 | 0.898 | 0.700 | 0.740 |
| Autoimmune thyroiditis | 0.829 | 0.419 | 0.187 | 0.728 |
| NVC (0-1 vs 2-3) | 0.181 | 0.501 | 0.284 | 0.124 |
| Dyspnea | 0.539 | 0.209 | 0.772 | 0.709 |
| Reflux | 0.078 | 0.123 | 0.870 | 0.748 |
| Dysphagia | 0.791 | 0.558 | 0.333 | 0.919 |
| Calcium antagonists | 0.513 | 0.643 | 0.156 | 0.329 |
| Iloprost | 0.203 | 0.161 | 0.318 | 0.319 |
| PGE | 0.299 | 0.414 | 0.606 | 0.596 |
| Sildenafil | 0.905 | 0.632 | 0.947 | 0.880 |
| MTX | 1.000 | 1.000 | 0.640 | 0.535 |
| MMF | 0.227 | 0.490 | 0.399 | 0.577 |

* p significative if <0.05

**Table S7. Significant correlations after adjusting for age and sex**

| ***Panel A (dichotomous variables)*** |  |  |
| --- | --- | --- |
|  | *SMA RI* | |
|  | β | *p* |
| ACA (yes vs no) | -0.32 | *0.006** |
| Scl70 (yes vs no) | 0.33 | *0.004** |
| DUs (yes vs no) | 0.26 | *0.038** |
| Diarrhea (yes vs no) | -0.21 | 0.078 |
|  |  |  |
|  | *SMA PI* | |
|  | β | *p* |
| ACA (yes vs no) | -0.18 | 0.11 |
| Scl70 (yes vs no) | 0.23 | *0.047** |
| Bosentan (yes vs no) | 0.28 | *0.027** |
|  |  |  |
|  | *IMA RI* | |
|  | β | *p* |
| Diarrhea (yes vs no) | -0.19 | 0.14 |
|  |  |  |
| ***Panel B (non-dichotomous variables)*** |  |  |
|  | *SMA RI* | |
|  | β | *p* |
| mRSS | 0.37 | *0.012** |
| FVC | -0.25 | 0.076 |
|  |  |  |
|  | *SMA PI* | |
|  | β | *p* |
| mRSS | 0.42 | *0.002** |
| FVC | -0.37 | *0.007** |
| PAPs | -0.31 | *0.024** |
| RRI dx | -0.11 | 0.45 |
| RRI sx | -0.18 | 0.21 |
|  |  |  |
|  | *IMA RI* | |
|  | β | *p* |
| GIT 2.0 incontinence | -0.32 | *0.012** |
|  |  |  |
|  | *IMA PI* | |
|  | β | *p* |
| GIT 2.0 incontinence | -0.30 | *0.020** |

* p significative if <0.05

**Table S8. Multivariate analysis of SMA RI and SMA PI**

|  | SMA RI  β p | | SMA PI  β p | |
| --- | --- | --- | --- | --- |
| mRSS | 0.248 | *0.030** | 2.995 | *0.004** |
| ACA | -0.189 | 0.161 | -0.313 | 0.756 |
| Bosentan | 0.400 | *0.003** | 3.508 | *0.001** |

* p significative if <0.05

SMA = superior mesenteric artery

IMA = inferior mesenteric artery

RI = resistive index

PI = pulsatility index

mRSS = modified Rodnan skin score

ACA = anticentromere antibodies
